# Supplementary material for: A Review of Evidence-Based Recommendations for Pericoronitis Management and a Systematic Review of Antibiotic Prescribing for Pericoronitis among Dentists: Inappropriate Pericoronitis Treatment Is a Critical Factor of Antibiotic Overuse in Dentistry
Source: Int J Environ Res Public Health. 2021 Jun 24;18(13):6796. doi: 10.3390/ijerph18136796 (PMC8296928; doi:10.3390/ijerph18136796)
Supplement: Supplementary file 1 [file ijerph-18-06796-s001.zip › ijerph-1237553-supplementary.pdf]

**Inclusion**
original article
scientific research reports
pericoronitis
antibiotic prescribing for pericoronitis
antibiotic prescription is given or can be calculated
in vivo study
human study
studies from 01/2000 to 05/2021

**Exclusion**
(n) of subjects < 15
study inclusion criteria for subjects (age, gender)
study inclusion criteria for subjects' condition (specific anamnesis)
in vitro studies
review articles
conference summaries
letters to the editor
case reports

Table S1. Inclusion and exclusion criteria.
